# Mixed reality for extraction of maxillary mesiodens

**DOI:** 10.1186/s40902-022-00370-6

**Published:** 2023-01-05

**Authors:** Yu Koyama, Keisuke Sugahara, Masahide Koyachi, Kotaro Tachizawa, Akira Iwasaki, Ichiro Wakita, Akihiro Nishiyama, Satoru Matsunaga, Akira Katakura

**Affiliations:** 1 Department of Oral Pathobiological Science and Surgery, Tokyo Dental College, 2-9-18 Kanda Misaki-Cho, Chiyoda-Ku, Tokyo, Japan; 2 Oral Health Science Center, Tokyo Dental College, 2-9-18 Kanda Misaki-Cho, Chiyoda-Ku, Tokyo, Japan; 3 Department of Anatomy, Tokyo Dental College, 2-9-18 Kanda Misaki-Cho, Chiyoda-Ku, Tokyo, Japan

**Keywords:** Mesiodentes, Image-guided surgery, Mixed reality (MR), Extraction

## Abstract

**Background:**

Mesiodentes are the most common supernumerary teeth. The cause is not fully understood, although proliferations of genetic factors and the dental lamina have been implicated. Mesiodentes can cause delayed or ectopic eruption of permanent incisors, which can further alter occlusion and appearance. Careful attention should be paid to the position and direction of the mesiodentes because of possible damage to adjacent roots in the permanent dentition period, errant extraction in the deciduous and mixed dentition periods, and damage to the permanent tooth embryo. To avoid these complications, we applied mixed reality (MR) technology using the HoloLens® (Microsoft, California). In this study, we report on three cases of mesiodentes extraction under general anesthesia using MR technology.

**Results:**

The patients ranged in age from 6 to 11 years, all three were boys, and the direction of eruption was inverted in all cases. The extraction approach was palatal in two cases and labial in one case. The average operative time was 32 min, and bleeding was minimal in all cases. No intraoperative or postoperative complications occurred. An image was shared preoperatively with all the surgeons using an actual situation model. Three surgeons used Microsoft HoloLens® during surgery, shared MR, and operated while superimposing the application image in the surgical field.

**Conclusions:**

The procedure was performed safely; further development of MR surgery support systems in the future is suggested.

## Background

Supernumerary teeth are teeth that are in excess of the normal number [1]. A mesiodens is a supernumerary tooth located in the maxillary central incisor region, and the overall prevalence of mesiodentes is between 0.15 and 1.9% [2–6]. If untreated, mesiodens is known to cause dental irregularities, delayed eruption of permanent teeth, late remnants of deciduous teeth, and cysts. Extraction of mesiodens is relatively common, and there are many opportunities for extraction at various ages from the deciduous period to the permanent dentition period. Mesiodens may cause damage to adjacent roots in the permanent dentition, errant extractions in the deciduous and mixed dentition periods, and damage to the permanent dental cup; therefore, careful attention is required.

Image-guided surgery utilizing augmented reality (AR) based on medical imaging is currently used in fields such as plastic surgery [7], orthopedic surgery [8], neurosurgery [9], and oral and maxillofacial surgery [10] and has been reported to be useful. The Microsoft® HoloLens comprises a head-mounted sensor camera, and spatial mapping of the surrounding environment enables the positioning of holographic images, thus achieving mixed reality (MR) with speech and/or gesture control. Several reports have now been published on the use of the Microsoft® HoloLens [11–13]. MR surgical support with holographic images superimposed on the patient enhances safety and reduces postoperative complications. Moreover, there have been reports about the feasibility of surgical training and remote surgical support [14], but this technique is yet to be used widely in oral and maxillofacial surgery.

MR technology has been applied as a strategy to avoid complications such as errant tooth extraction and root damage. The present report describes three cases in which MR surgical support using Microsoft® HoloLens was used in the extraction of mesiodens. This technique enabled surgery that was safe and in accordance with the preoperative planning, and this report details this technique, including the preoperative preparation protocol.

## Methods

### *Preoperative preparation (Microsoft*.*® HoloLens application)*

The direction of mesiodentes extraction was chosen using CT data obtained preoperatively with the SOMATOM Definition AS (Siemens, Forchheim, Germany).

**Fig. 1:**
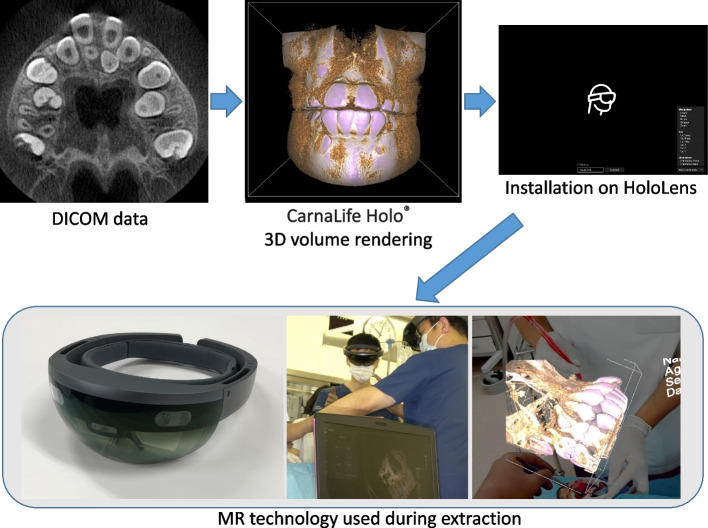
Preoperative preparation and MR technology used during extraction

The CarnaLife Holo® (MedApp, Kraków, Poland) was used to read Digital Imaging and Communications in Medicine (DICOM) data and display 3D volume rendering data, and a hologram was projected onto the surgical field by connecting a Microsoft® HoloLens (Fig. 1). The present study was approved by the ethics committee of Tokyo Dental College (Tokyo, Japan; no.794, 1054).

## Results

### Cases

The patients ranged in age from 7 to 11 years, all three were boys, and the direction of eruption was inverted in all cases. The extraction approach was palatal in two cases and labial in one case. The average operative time was 32 min, and bleeding was minimal in all cases. No intraoperative or postoperative complications occurred (Table 1). Two representative cases are described in detail below.

**Table 1:** Clinical information

Case	Age	Sex	Number of mesiodens	Approach of extraction	Operation time (min)	Complication
1	9	Male	2	Palatal	47	No
2	11	Male	1	Labial	19	No
3	7	Male	2	Labial	30	No

### Case 1

**Fig. 2:**
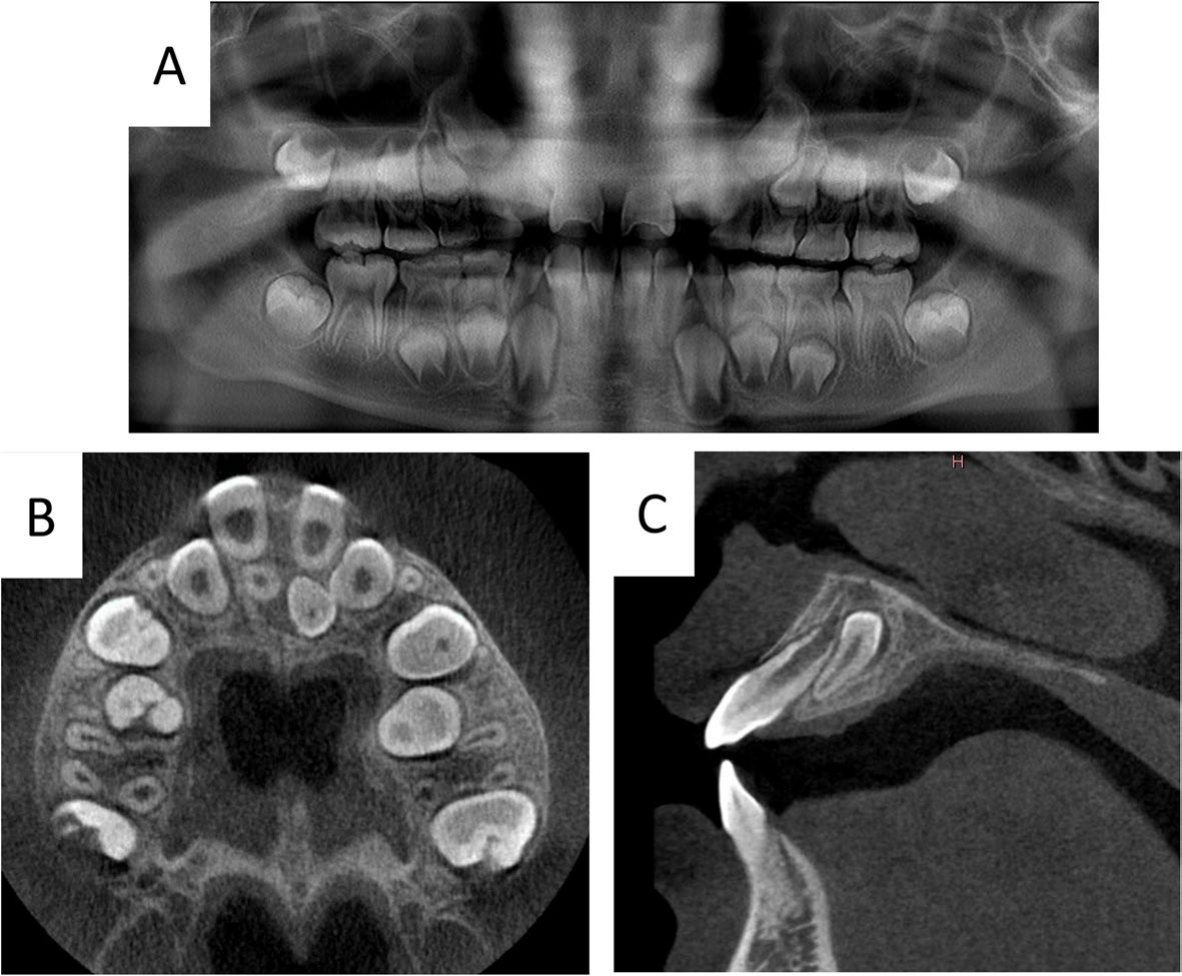
Case 1. **A** Panoramic radiograph. **B** CBCT axial view. **C** CBCT sagittal view

A 9-year-old boy presented with his mother to the Department of Oral and Maxillofacial Surgery, Tokyo Dental College Suidobashi Hospital, with a chief complaint of malocclusion, as stated by his mother. His medical history was noncontributory. The intraoral examination showed median eminence of the permanent maxillary central incisor. A panoramic radiograph was taken (Fig. 2A), showing two inverted supernumerary teeth on the palatal side, which were better observed in the occlusal view. CT was performed with a large field of view. The CT showed that the excess tooth was in contact with the root of the permanent tooth for the most part (Fig. 2B, C).

**Fig. 3:**
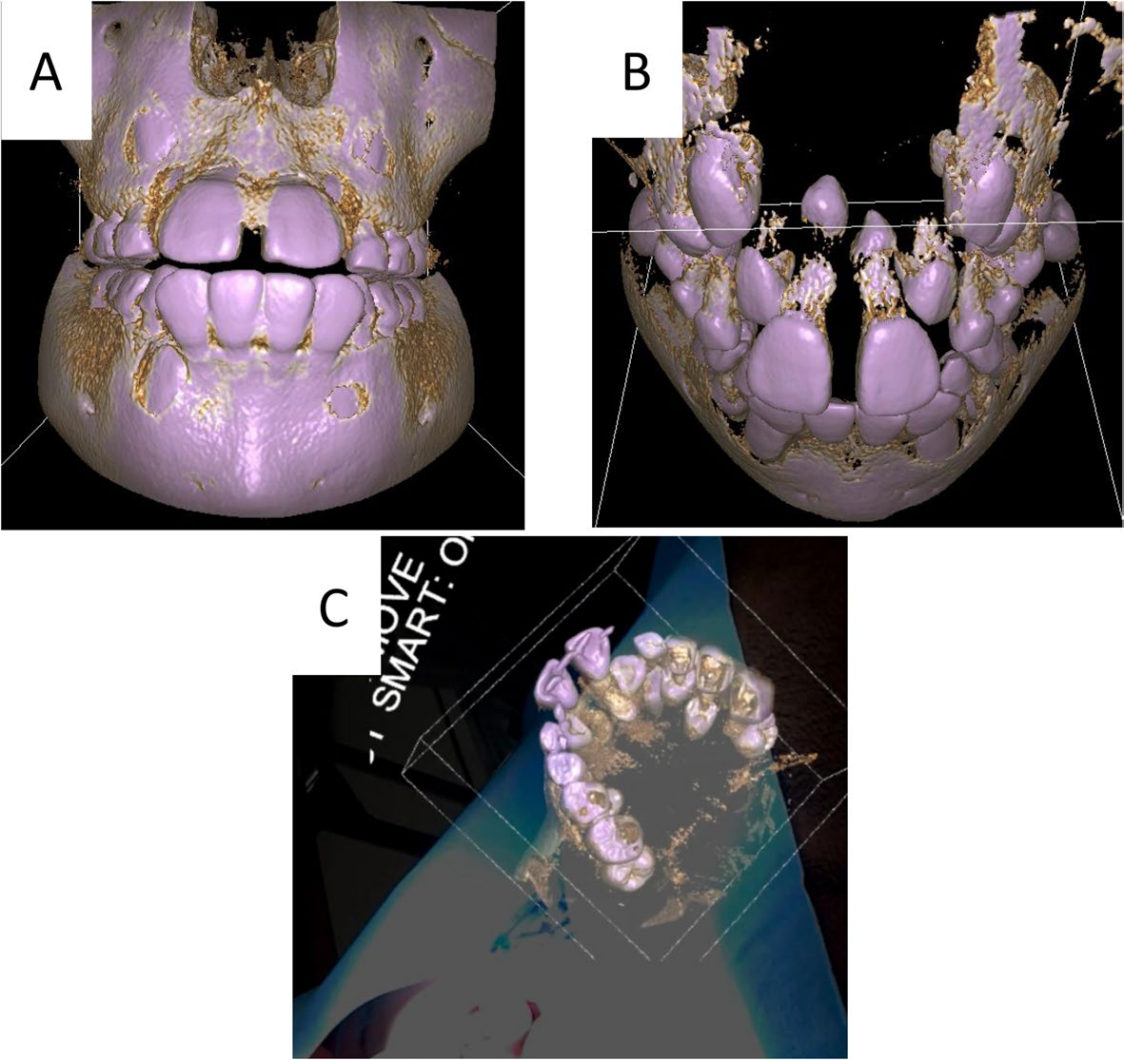
Case 1. **A** 3D constructed image. **B** 3D volume rendering. **C** Intraoperative view

In order to avoid errant extractions and damage to the permanent crown, 3D volume rendering data was created from the preoperative CT DICOM data (Fig. 3A, B). Under general anesthesia, the tooth was extracted from the palatal side (Fig. 3A, B). It was possible to perform surgery while placing the hologram in the indirect field of surgical view. The operative time was 47 min. The procedure went well, and at the follow-up visit 2 weeks later, progressive healing of the soft tissues was observed. At the 1-year post-surgery clinical examination, complete healing of soft tissues and a crowded dentition were observed.

### Case 2

**Fig. 4:**
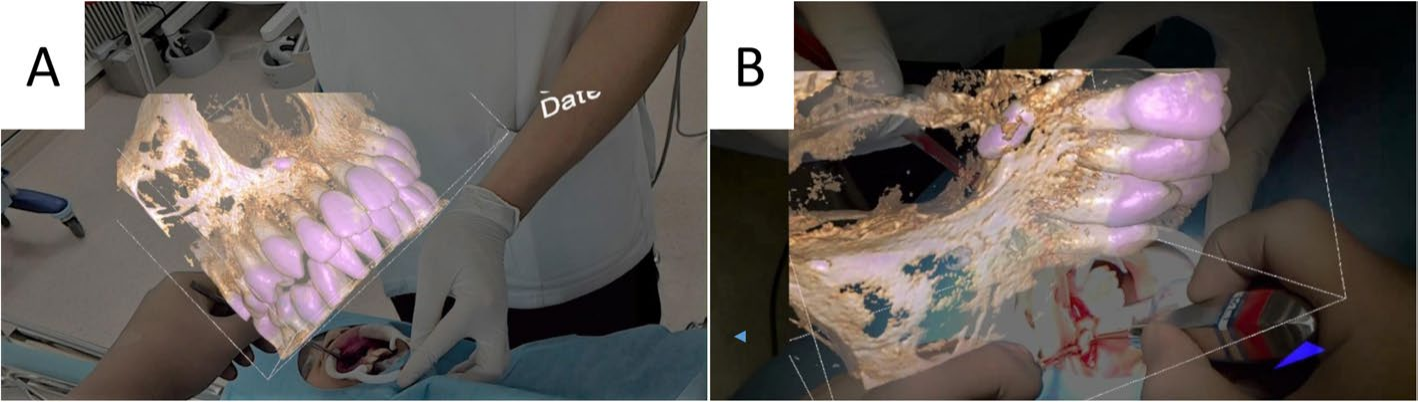
Case 2. **A**, **B** Intraoperative view using MR technology

An 11-year-old boy presented with malocclusion to his family dental clinic. Panoramic radiographs were taken, showing one inverted supernumerary tooth. He was referred to our hospital and had a cone beam CT (CBCT) taken. A 3D volume rendering image showed the appearance of the supernumerary tooth crown piercing through the nasal fossa floor (Fig. 4). We decided to approach from the labial side and to extract the tooth from the nasal side. His medical history was noncontributory. An incision was made in the gingivobuccal transition area and dissected to reveal the anterior nasal spine and the inferior margin of the pyriform foramen. While confirming the projected hologram, minimal osteotomies were made, the tooth crown was clearly indicated, and the tooth was extracted. The operative time was 19 min. No postoperative complications, such as abnormal upper lip morphology or nose bleeding, occurred.

### Case 3

**Fig. 5:**
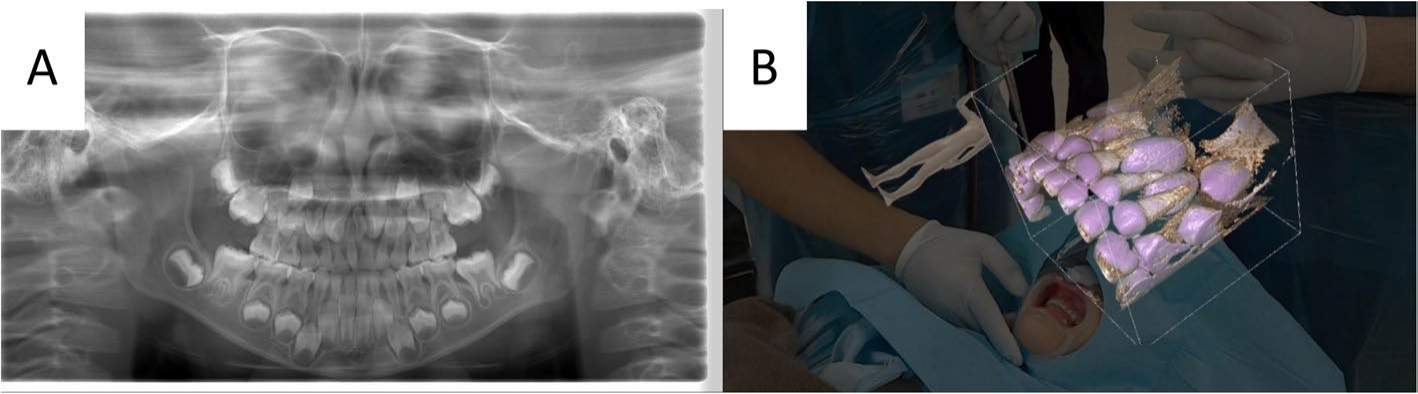
Case 3. **A** Panoramic radiograph. **B** Intraoperative view using MR technology

A 7-year-old boy presented with malocclusion to his family dental clinic. Panoramic radiographs were taken, showing two supernumerary teeth. He was referred to our hospital and had a CBCT taken. A 3D volume rendering image showed the one erupted supernumerary tooth in between A┴A and another inverted supernumerary tooth in between 1┴1. We decided to approach from the labial side and to extract the teeth. His medical history was noncontributory. We made a cervical incision to reveal the supernumerary teeth. While confirming the projected hologram, minimal osteotomies were made, and the teeth were extracted (Fig. 5A, B). The operative time was 30 min. No postoperative complication such as erroneous tooth extraction and damage to adjacent roots occurred. This operation was performed by a young dentist in 2 years of post-graduation with a senior doctor.

## Discussion

It has been reported that when supernumerary teeth are classified by location, they occur most frequently in the maxillary incisor region, followed by the maxillary molar region, and then the mandibular molar region [1], and mesiodentes (supernumerary teeth of the mandibular incisor region) are frequently encountered in routine clinical practice. Various causes have been suggested, including over-proliferation or prolonged survival of dental lamina epithelial cells [15, 16], and division of dental lamina [15], but the precise etiology of mesiodentes is uncertain. The male to female ratio of people developing mesiodentes is 2:1, and they are most frequently aged 10 to 20 years [17], which is consistent with the period of eruption and replacement of the front mandibular teeth. X-radiography is performed as a screening for delayed eruption and rotated, displaced, and missing teeth, and mesiodentes are frequently detected as a result of this.

Panoramic and intraoral X-radiography is currently used for the diagnosis of maxillary central supernumerary impacted teeth. The obstructive shadow of the cervical vertebrae makes it difficult to examine the front maxillary teeth using panoramic X-radiography, and although intraoral X-radiography (which has been in previous use) has a high resolution, in patients with overlapping impacted and permanent teeth, observation is difficult, and there are also problems with interpretation of the spatial relationships between the nasopalatine duct and nasal lumen. X-radiographic CT has recently been reported as useful for diagnostic imaging of supernumerary impacted teeth [18]. We use panoramic and/or intraoral X-radiography for the diagnosis of maxillary central supernumerary impacted teeth and to guide decisions regarding the appropriate treatment. In addition, in principle, we use CT for patients with unerupted teeth with whom the buccopalatal position cannot be ascertained by palpation. By these means, it is possible to accurately confirm the 3D position of impacted teeth, decide upon the approach for tooth extraction, and prevent postoperative complications. In addition, there have been cases of multiple supernumerary teeth that are not detected on the previous X-ray, but are detected for the first time by CT. It is important to perform preoperative CT in order to determine the 3D position of the teeth and to decide upon the approach for extraction. However, direct, intraoperative verification of the surgical field is not feasible, and accidents such as mistaken extraction and loss of permanent teeth are not uncommon.

The Microsoft® HoloLens has a head-mounted sensor camera, and spatial mapping of the surrounding environment enables the positioning of holographic images and the use of MR with speech and/or gesture control. Although there have been previous reports of the use of Google Glass as a method for applying AR technology in the operating theater [19, 20], one disadvantage that has been put forward is the inability to perform hands-free operations. In addition, the Oculus Quest is a tool that can be used to apply VR technology; however, because the hologram is projected into a virtual space, real-world projection is not feasible, and this method cannot be used for intraoperative surgical support and is instead used for preoperative simulation and education [21–23]. Surgical support using the HoloLens is characterized by being hands-free, and it enables the projection of clinical data [11–14]. We have previously reported that MR surgical support with superimposed holographic images enables confirmation of the 3D position and anatomical morphology of the maxilla, which is expected to improve surgical safety and reduce postoperative complications [12]. Oral and maxillofacial surgery using HoloLens has been reported for impacted teeth extraction [11], tumor resection [12, 24], orthognatic surgery [13], cystectomy [11, 25], and mandibular reconstruction [26]. In addition, there have been reports of the use of HoloLens for teaching root canal treatment [27]. So it is expected to be applied in dental offices and hospitals in the future.

HoloLens projects pre-prepared CT data onto the surgical field, so the surgical time is not prolonged by the setting time. A simulation-based study reported that the use of HoloLens shortened the operation time in the endoscopic surgery [28]. In addition, there is no increase in the cost to the patient. By making it possible to display various information on the surgical field by the HoloLens, oral surgeons will be able to refer to various information without moving their field of view, which will allow them to perform safer and more accurate surgery. On the other hand, MR surgery has many limitations. Firstly, in MR surgery, there is difficulty in the manual superimposition of the VR onto the patient. Secondly, MR surgery is not able to perform a virtual operation of holograms intraoperatively. Finally, using HoloLens, patients feel weight and fatigue. The weight of HoloLens is 579 g, and the measures are approximately 9 × 26 cm. In 2019, the Microsoft® HoloLens2 was released. The weight is 566 g and the measures are approximately 13 × 30 cm. Although there are no significant changes in size, the weight distribution had been changed to improve weight perception and to reduce for feeling fatigue. In addition, there was concern about the darkening of the field of view during surgery with Hololens, but this was not a concern under the lights of the operating room.

In the present study, a 3D image was constructed from CT data obtained preoperatively and was projected as a hologram. Maxillary central supernumerary tooth extraction was then performed. The first advantage of this method is that it does not require movement of the surgical field and the second advantage is that the target tooth and adjacent teeth can be viewed clearly by adjusting the hologram. As a result of the first advantage, even if the jawbone has numerous impacted teeth, as observed during the mixed dentition period, the potential for mistaken tooth extraction is reduced. The second advantage means that when impacted teeth are displayed, the risk of loss of the roots of adjacent teeth due to bone drilling can be expected to be reduced. In the present study, no accidental problems such as root loss or mistaken extraction occurred. Even young dentists were able to perform extractions safely. Therefore, it is considered that MR technology using the HoloLens is useful for techniques requiring a great deal of care, such as extraction of maxillary supernumerary impacted teeth.

## Conclusions

In this report, incorporation of MR technology during preoperative confirmation and intraoperative visualization of the oral and maxillofacial region enabled us to achieve safe extraction. This MR technology has the potential for performing safe and effective surgical procedures for other conditions and we intend to investigate this in the near future.

## Data Availability

The analyzed data sets generated during the present study are available from the corresponding author on reasonable request.

## Abbreviations

3D: Three-dimensional
CAD/CAM: Computer-aided design/computer-aided manufacturing
MR: Mixed reality
AR: Augmented reality
CT: Computed tomography
